# Delaying vascular aging: A new prospect in medicine

**DOI:** 10.17179/excli2019-1854

**Published:** 2019-12-13

**Authors:** Ayesha Jamil, Sana Habib

**Affiliations:** 1 Fatima Jinnah Medical University, Lahore, Pakistan; 2 Dow University of Health Sciences, Karachi, Pakistan

## ⁯

**Dear Editor,**

Aging is a physiological process that is inevitable for all human bodies. By 2030, it has been estimated that almost 20 % of the population will be aged 65 years or older and cardiovascular diseases (CVD) will result in 40 % of all deaths in this demographic (North and Sinclair, 2012[5]). Likewise, cardiovascular disease is more likely to be seen in accelerated aging syndromes, such as Hutchinson-Gilford progeria syndrome (HGPS), which further cements the fact that vascular aging is leading to a rise in CVD (Capell et al., 2007[1]). Therefore, comprehending the intricate processes underlying the age related changes to the cardiovascular system is of utmost importance.

The effect of aging on cardiovascular health is in part because aging perturbs a number of metabolic and hemodynamic mechanisms in the cardiovascular system in general and the vascular endothelium in particular. Aging affects the vasculature by causing both structural and functional changes via increasing oxidative stress, premature cellular senescence and notably impairing the synthesis and release of endothelium-derived vasoactive molecules (Ghebre et al., 2016[2]). This leads to the release of harmful substances from the aging endothelium, which accelerates DNA damage, telomere erosion, senescence and stiffening of the vessel wall making it susceptible to develop hypertension, atherosclerosis and other risk factors for cardiovascular disease. 

Since the aging process is inescapable and will end up burdening healthcare in the not so distant future, researchers are striving to find ways to prevent aging in the first place. Prolonged periods of starvation leads to the formation of ketone bodies (KB), consisting of beta hydroxybyutarate (β-HB), which has shown to be therapeutic against neurological diseases (Paoli et al., 2014[6]). Hence, the notion of ketone bodies preventing vascular aging can be a lucrative research venture which may ultimately lead to change in disease patterns of the geriatric population.

However, the method by which KB acts on the vascular system and on the aging process is largely unknown. The studies ave shown that β-HB might promote vascular cells quiescence. This predominantly inhibits both stress-induced premature senescence and replicative senescence via p53 independent mechanism. Furthermore, a heterogenous nuclear ribonucleoprotein (hn RNA A1) has been identified to have a direct binding site for β-HB. Binding of β-HB to its target site (hn RNA A1) significantly increases hn RNP A1 binding with octamer binding transcriptional factor (Oct)4 mRNA, enhancing stabilization of Oct4 mRNA and Oct4 expression. The overall result is an increase in Lamin B1, a key-factor against DNA damage-induced senescence. Researchers have also found that as senescent cells become unable to multiply and divide, β-HB in addition to promoting cell division can also prevent these cells from getting old (Han et al., 2018[3]).

Recently, a new dietary regimen is being adopted, popularized as the 'ketogenic diet'(KD), which comprises of high fat and low carbohydrate food. This is set to replicate the effects of prolonged calorie restriction. The short term effects include the loss of body weight and an increase in KB production by the liver. The long term effects on cardiovascular system remain unknown (Sedej, 2017[7]).

Nonetheless, due to the fast and excessive loss of body fat, deleterious side effects of KD include menstrual abnormalities and osteoporosis in females. A β-HB mimetic would help in the long term, as it would not require the vigorous loss of body fat nor a tight diet control. But the production of such a drug is contingent on the fact that we are still unsure about how ketone bodies exert its protective effect on the vasculature (Martin et al., 2006[4]). Considering that there is a dearth of research regarding long term effects of calorie restriction, ketogenic diet and a β-HB therapeutic mimetic, there should be a renewed clinical interest in the modification of the human diet and preventive medicine, as we are advancing into the new decade of the millennia with an evolving elderly population.

## Conflict of interest

The authors declare no conflict of interest.
